# Study on the Structure–Activity Relationship and Oil Displacement Characteristics of the Polysurfactant Agent

**DOI:** 10.3390/polym16030383

**Published:** 2024-01-30

**Authors:** Jingang He, Lin Yuan, Bicheng Gan, Zhiqiang Liu, Haixiang Zhang

**Affiliations:** 1 No. 1 Oil Production Plant, Daqing Oilfield Company Limited, PetroChina, Daqing 163000, China; yuanlin_a@petrochina.com.cn; 2 Development Division of Daqing Oilfield Co., Ltd., Daqing 163000, China; 3 School of Petroleum Engineering, Northeast Petroleum University, Daqing 163000, China; gbicheng@foxmail.com (B.G.); zhiq.liu@foxmail.com (Z.L.); haixiangzhang1225@163.com (H.Z.); 4 Nepu Sanya Offshore Oil&Gas Research Institute, Northeast Petroleum University, Sanya 572024, China

**Keywords:** polymer surfactant, microscopic morphology, emulsifying behavior, regulating drive plugging, enhanced oil recovery

## Abstract

This study examines a versatile polymer known as polysurfactant, which is synthesized by co-polymerizing flexible acrylamide and sodium acrylate hydrocarbon chain. The polymer serves as a backbone and possesses active functional groups. Notably, the polysurfactant exhibits superior plugging and flooding abilities compared to conventional polymers. The primary objective of this paper is to investigate the properties and oil displacement characteristics of the polysurfactant through indoor physical simulation experiments. The results demonstrate that the multi-branched structure of the polysurfactant enhances its ability to associate, leading to the formation of a unique spatial network structure. The inclusion of multi-branched structures notably amplifies the association effect. The critical concentration for the association is estimated to be around 800 mg/L, at which juncture the polysurfactant exhibits a viscosity retention rate surpassing 90% subsequent to shearing. Furthermore, this spatial network structure exhibits self-recovery capabilities after experiencing shear failure and displaying strong viscosity and shear resistance. In addition, the concentration of the polysurfactant can control the hydrodynamic feature size, which shows its adaptability in regulation and oil-repelling functions at reservoir permeabilities ranging from 500 to 2000 × 10^−3^ μm^2^ with resistance coefficients ranging from 108 to 320. During the microscopic oil displacement process, the polysurfactant exerts a significant impact on mobility control, while the elastic pull clearly demonstrates a commendable viscoelastic oil displacement effect. The polysurfactant exhibits a specific degree of emulsification capability towards crude oil, leading to the emulsion exhibiting typical pseudoplastic fluid characteristics. The utilization of emulsification transportation and emulsification blockage contributes to the enhancement of oil recovery. As a result, the polysurfactant exhibits multifaceted capabilities, encompassing profile control, flooding, and plugging, owing to its unique structural characteristics. Through the implementation of a field test focused on flooding in the Daqing Oilfield, a significant enhancement in the recovery rate of 10.85% is observed, accompanied by a favorable input–output ratio of 1:3.86, thereby generating significant economic advantages.

## 1. Introduction

Despite the gradual rise in the production of unconventional oil and gas resources, conventional subsurface methods remain the primary approach for oil and gas production [1,2]. Polymer flooding technology is widely recognized as the key technology for attaining elevated and consistent production levels in the Daqing Oilfield [3,4,5,6,7]. With the advent of the post-polymer flooding era, there is an increasing demand for enhanced oil recovery (EOR) technology and displacement agents to ensure the long-term sustainability of the Daqing Oilfield [8]. Although high-concentration polymer demonstrates notable capabilities in expanding the sweep volume, its ability to enhance oil washing efficiency is constrained despite its elastic properties. The implementation of Alkaline/Surfactant/Polymer (ASP) flooding is aimed at simultaneously increasing the swept volume and improving the efficiency of oil washing. However, the combined impact of chemical interactions, on-site management difficulties, and the associated high expenses present obstacles to the advancement of ASP flooding beyond polymer flooding [9,10]. As a result, the exploration of a multifunctional single agent emerges as a promising avenue for enhancing oil recovery following polymer flooding.

The implementation of Alkaline/Surfactant/Polymer (ASP) flooding is aimed at simultaneously increasing the swept volume and improving the efficiency of oil washing. However, the combined impact of chemical interactions, on-site management difficulties, and the associated high expenses present obstacles to the advancement of ASP flooding beyond polymer flooding. As a result, the exploration of a multifunctional single agent emerges as a promising avenue for enhancing oil recovery following polymer flooding [11]. The polysurfactant molecule, derived from acrylamide and modified polymer, incorporates functional groups such as amide and alkyl sulfonic acid. Upon dissolution in water, the resultant solution exhibits a resilient three-dimensional network structure, effectively capturing crude oil and impeding the merging of oil droplets [12,13,14]. The principal mechanisms through which the polysurfactant augments oil recovery encompass viscoelasticity, emulsification, solubilization, and the regulation of the oil-water mobility ratio. The injection performance of polysurfactant flooding surpasses that of ordinary polymer flooding, and it possesses a resilient profile adjustment effect that improves the flow of crude oil in thin and poor reservoirs [15,16,17]. Field tests have provided evidence that polysurfactants yield higher rates of enhanced oil recovery compared to other innovative polymers [18,19,20,21]. However, the application of polysurfactants in specific low-permeability reservoirs also presents certain challenges. The presence of a highly extensive molecular network structure in an aqueous solution obstructs select low-permeability pores and increases the viscosity of the displacement fluid, resulting in a decrease in reservoir permeability. Therefore, it is necessary to introduce specific chemical agents to mitigate the aforementioned plugging phenomenon [22]. Therefore, it is crucial to undertake further investigation into the correlation between the structure and activity of the polymeric surfactants, as well as the mechanism by which they displace oil. This research aims to clarify the characteristics of oil displacement and the potential applications of these surfactants, ultimately providing a theoretical foundation for their extensive implementation and utilization through field experiments.

This study utilizes the integration of microfluidic technology and microfluidic experimental equipment to investigate the mechanism of oil displacement. The micro-forming system enables the observation and recording of microscopic pore throats, as well as the visual and convenient examination of fluid migration in formation pores. Consequently, this approach offers a robust theoretical foundation for comprehending the mechanisms of various oil displacement systems. Notably, the microfluidic technology exhibits remarkable advantages in terms of visualization and controllability [23,24,25,26]. Laboratory experiments were conducted to investigate the micro-morphology, viscosity enhancement, shear stability, and water molecular dynamic characteristics of three types of polymers: polymer surfactant, medium polymer, and salt-resistant polymer. The findings revealed that the presence of diverse functional groups in the monomer significantly improved the interfacial activity of the polymer surfactant. Moreover, the polymer exhibited superior viscosification shear resistance compared to the other two polymers at the critical association concentration. The polymer surface agent exhibits enhanced viscosity adjustment capabilities through minor concentration adjustments, resulting in significant savings in polymerization volume. Additionally, the polymer surface agent demonstrates adaptability through single-dose adjustment, plugging, and displacement, achieved by gradually reducing its concentration. Investigation into the micro-displacement characteristics of the polymer surfactant reveals that the emulsification effect of the surfactant contributes to improved oil recovery rates through emulsification transport and sealing. A field test was conducted in the eastern region of the North First District of the Daqing Oilfield to evaluate the effectiveness of polymer surface agent flooding following polymer flooding. The study focused on examining the exceptional characteristics of the polymer surface agent. Macroscopically, the polymer surfactant has the most significant effect on improving the diversion rate of low-permeability layers, and at the microscopic level, it exerts a stronger viscoelastic oil displacement effect. The emulsification type dominated by O/W can further improve oil recovery after polymer flooding through emulsification carrying and emulsification plugging effects.

## 2. Experimental Details

### 2.1. Experimental Material

**Figure 1:**
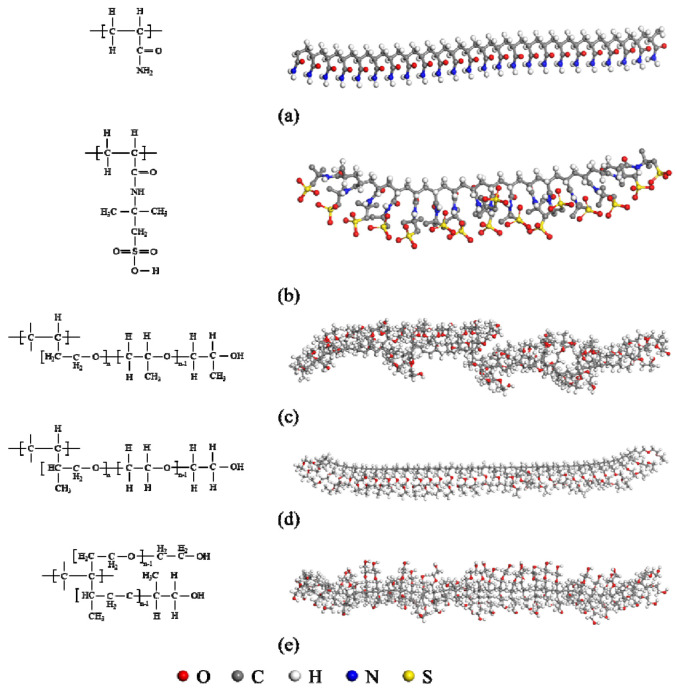
The initial configuration and structural formula of the structural unit of the polysurfactant. (**a**–**e**) are the monomers that make up the surface activator, respectively.

The polysurfactant (Daqing Oilfield, Daqing, China) used in the experiments was synthesized in the laboratory, and the molecular structures are shown in Figure 1. Mid-split polymers [27] (Hualong Xiang Chemical, Daqing, China) are produced by Hualong Xiang Chemical and have a molecular weight of 8 million daltons. Salt-resistant polymers [28] (Daqing Oilfield, Daqing, China) are commercial polymers supplied by the Daqing Oilfield and have a molecular mass of 700 × 10^4^.

### 2.2. Polymer Microscopic Morphology Observation Experiment

The polysurfactant, mid-split polymers, and salt-resistant polymers underwent individual testing using the GeminiSEM300 (ZEISS, Baden-Wurttemberg, Germany.) field emission scanning electron microscope. The polymer samples were prepared through freeze-drying, wherein the samples designated for testing were deposited onto pristine silicon wafers, rapidly frozen using liquid nitrogen, maintained in a frozen state, and subsequently subjected to freeze-sublimation in a freeze-dryer for approximately 72 h. Following the complete drying of the samples, a small quantity of dried samples was subjected to gold film spraying, enabling the examination of the aggregation morphology of the polymers under a scanning electron microscope. Throughout the observation procedure, electron excitation of 5 Ke was employed to capture images of the microscopic morphology of the polymers at varying magnifications.

### 2.3. Hydrodynamic Characteristic Size Test of Polymer

Experiments were conducted on three polymer systems, employing the principle of measuring hydrodynamic characteristic size. The polymer systems encompassed salt-resistant polymers and mid-separation polymers. The concentration ranges of the three agents spanned from 200 mg/L to 3500 mg/L. The standard for viscosity retention rate following shearing of an ordinary middle polymer was established at approximately 60%. Subsequent tests determined the shearing condition to be 20 s at the 2nd gear of the Wu Yin agitator. Acquire a sample of the diluted target liquid and utilize a Brookfield viscometer to evaluate the viscosity at a rotational velocity of 6 revolutions per minute (rpm). After achieving a stable measurement, typically after a minimum duration of 10 min, document the reading. Subsequently, introduce the target liquid into a stirrer and expose it to shear forces. Once any bubbles generated by the shearing process within the solution have been eliminated, obtain a sample and reassess the viscosity.

The experiment was carried out under standard atmospheric conditions, with the experimental setup connected and a pressure of 0.1 MPa applied using a gas cylinder. The filter container was filled with a 200 mL solution, and the filter membrane was gradually reduced from an initial size of 3 to a final size of 0.15. Each filter membrane allowed a volume of 20 mL of solution to pass through. The viscosity of the filtrate was measured using a Brookfield viscometer, and a graph illustrating the relationship between the viscosity retention rate and the pore size of the filter membrane was plotted. The inflection points observed on the curve indicate the hydrodynamic dimensions of the polymer. The filter membrane size that aligns with the inflection point of the viscosity retention curve represents the hydrodynamic characteristic size of the polymer at this particular concentration.

### 2.4. Emulsion Configuration

In an Erlenmeyer flask with a stopper, a 100 mL volume is designated for the measurement of 15.00 g of the surfactant mother solution, which has been prepared using saline. Subsequently, 85.00 g of salt water is to be added, and the resulting mixture is to be placed on a magnetic stirrer. The stirring process should be conducted for a duration of 15 min, leading to the formation of a surfactant solution with a concentration of 0.3% per 100 g. Following this, the liquid tube of the emulsifier is to be used for the measurement of 10 g of crude oil (measured to the nearest 0.01 g) and 40 g of the aforementioned surfactant solution (measured to the nearest 0.01 g). To initiate the operation of the emulsifier, activate the circulating constant temperature water bath and adjust the temperature settings to attain the desired molding temperature. Preheat the system for a duration of 30 min. Subsequently, regulate the velocity of the piston on the emulsifier panel to 2 m/min, activate the motor, and commence the emulsification process for a period of 1 h.

### 2.5. Experimental Method of Polymer Flooding

**Figure 2:**
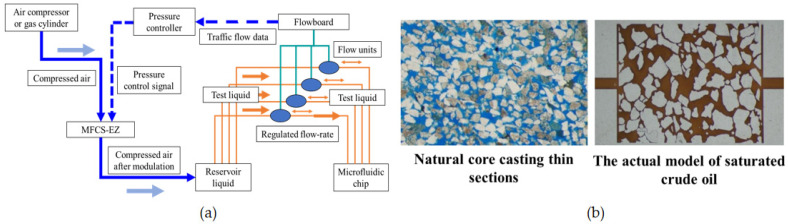
Microfluidic experimental device and microfluidic chip: (**a**) Microfluidic experimental device; (**b**) Natural core casting thin sections and microfluidic chips.

The emulsion utilized in the experiment was produced using a homogenizing apparatus. The water-to-oil ratio was 9:1. The emulsifier’s circulating water bath, set at a constant temperature, was activated and calibrated to attain the desired molding temperature and subsequently preheated for a duration of 30 min. The speed of the piston on the emulsifying machine’s control panel was adjusted to 2 m/min, the motor was initiated, and emulsification was carried out for a period of 1 h. The experimental instrument was connected as depicted in Figure 2a. The microfluidic chip (Daqing Oilfield, Daqing, China) utilized in this study was obtained from the cast core extracted from the Daqing Oilfield formation, as illustrated in Figure 2b. The microfluidic chip was saturated with crude oil and subsequently filled with water under a constant pressure of 200 mBar until the swept volume ceased to expand. The pressure was then incrementally raised to 300 mBar, and the polymer was filled with water until the swept volume no longer exhibited any further expansion. This process was repeated by increasing the pressure in increments of 100 millibars until reaching a final pressure of 600 millibars. The preceding steps were reiterated, with the flow sensor (Buekert, Bautzen, Germany.) and photography employed to document the alterations in flow and oil distribution throughout the procedure. Subsequently, the enhanced oil production was determined using image recognition technology. At the same time, the dynamic variations of emulsion droplets at the pore throat were observed and photographed to ascertain the principles governing retardation and retention, as well as their role in enhancing oil recovery. During the whole process, the liquid production and pressure data of each layer will be carefully recorded. Since the temperature of the Daqing oil layer is about 45 °C, experiments must be carried out at this temperature to obtain the best experimental results.

## 3. Results and Discussion

### 3.1. Characterization and Micromorphology of Polymers

The three polymer systems exhibit comparable molecular backbone compositions, albeit with distinct monomers for graft copolymerization. The polymeric surfactants and salt-resistant polymers possess elevated levels of sulfur content, with the former exhibiting higher sulfur content than the latter. Their primary function entails enhancing the temperature and salt resistance of the chemical flooding system. The elemental analyses of the three polymers revealed that the polymeric surfactants exhibited a notable elevation in nitrogen content, indicating the presence of cationic hydrophobic monomers. The disparity in elemental composition between the polymeric surfactants and salt-resistant polymers lies in the total carbon number (carbon-hydrogen ratio). The polymeric surfactants possess a higher carbon-to-hydrogen ratio, a greater number of monomer molecules, and, consequently, a more advanced grafted structure.

The presence of diverse functional groups in the monomer enhances its interfacial activity and reveals distinct small molecule fragment bodies and multi-branched structures, thereby resulting in an association effect. The dissimilarity in element composition and molecular structure between the polysurfactant and the salt-resistant polymer and middle polymer accounts for the noticeable discrepancies in their mi-ecomorphological and oil displacement characteristics.

**Figure 3:**
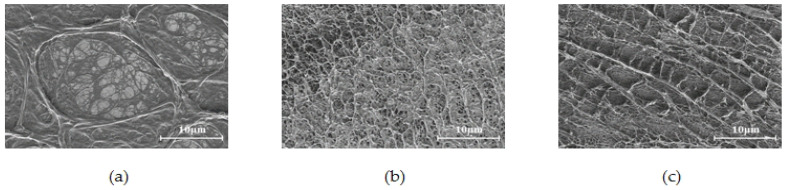
Three polymer microstructures: (**a**) Polymeric agent; (**b**) Medium polymer; (**c**) Salt-resistant polymer.

The microscopic morphology of the polymer serves as a perceptible manifestation of its molecular structure. By examining the microscopic molecular morphology of the polysurfactant, medium polymer, and salt-resistant polymer under identical concentration and drying conditions, one can discern the disparities in their molecular chain and spatial structure. Figure 3 illustrates the scanning electron microscopy images (ZEISS, Baden-Wurttemberg, Germany.) depicting the microscopic morphology of various polymer types.

Among the various constituents, the polysurfactant’s molecular chain stands out as the most resilient, exhibiting a substantial network configuration in its conformation. Additionally, a complex ribbon-like structure of smaller chains is interspersed within the network, as depicted in Figure 3a. This extensive network structure possesses spatial dimensions and lacks a discernible primary chain, signifying its characteristic as a spatial body structure. Through analysis using the nuclear magnetic hydrogen spectrum, it is determined that the molecular chain is formed through the association of small molecules, thereby augmenting its length. The polymer located in the middle possesses a network structure resembling a sheet characterized by a fine and uniform molecular chain skeleton. This structure exhibits a prominent long straight chain configuration, forming a spatial arrangement through the winding of these long chains, as depicted in Figure 3b. In contrast, the salt-resistant polymer displays a distinct, long, straight main chain structure, characterized by a robust main chain with significant rigidity and the presence of a certain number of side chains. This particular arrangement makes it challenging for the polymer to intertwine and curl, unlike its neutral counterpart. The dependence of spatial structure formation on side chain cross-linking is evident in Figure 3c. Table 1 presents the comparison of spatial structures among the three polymer types. The structural attributes of the polysurfactant confer it with a robust capability to regulate the mobility of sizable pores during association conditions. Furthermore, its short-branched structure allows for entry into the narrow pore channel for displacement and cleansing upon disassociation. And its concentration of the substance is utilized to regulate the incidence of association conditions, thereby exhibiting adaptive plugging adjustment properties in various permeability reservoirs when assuming the roles of association and disassociation.

### 3.2. Thickening Properties and Shear Stability of Polysurface Agents

**Figure 4:**
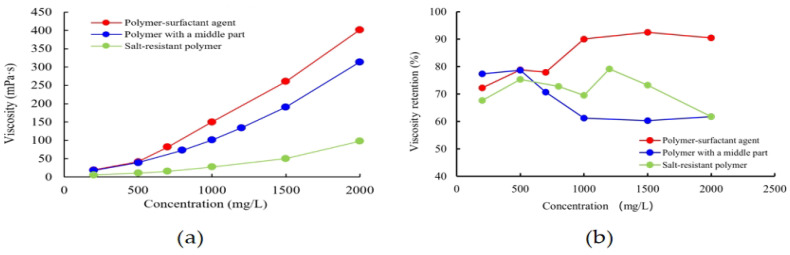
Viscosity-enhancing properties and post-shear viscosity retention of three polymers: (**a**) Viscosification properties of three polymers; (**b**) Viscosity retention rate of three polymers after shearing.

The augmentation of viscosity is a crucial element in polymers for the purpose of regulating flow [29,30,31,32]. This is accomplished by means of the interlinking of polyepitaxials, which possess distinctive branched structural attributes, leading to the creation of a comprehensive network structure. As a result, this network structure heightens the impediment to intramolecular transport. The viscosity of the polyepitomizer solution demonstrates a substantial discrepancy between pre- and post-association, as illustrated in Figure 4a. Furthermore, the process of association, under different concentration conditions, exerts a direct influence on the percolation behavior at the critical point. Upon comparing the viscosity of the three distinct polymer types, it becomes evident that the viscosity remains relatively stable below a concentration of 800 mg/L. Nevertheless, once the concentration exceeds 800 mg/L, the viscosity of the poly-surfactant undergoes a sudden surge, signifying its hydrophobic nature. This implies that the polymer’s critical concentration is determined to be 800 mg/L. Moreover, the viscosity of the polymers within the concentration range of 1000–2000 mg/L undergoes substantial variations, characterized by a gradual increase and deceleration in viscosity as the concentration of the poly-surfactant solution continues to rise. The rate of increase slows down as the concentration of the polyepitaxial solution increases while its viscosity steadily intensifies. Once the concentration exceeds 1500 mg/L, the solution exhibits non-homogeneous properties, resembling a translucent substance. The performance of the salt-resistant polymer falls within the range of polyepitaxanthin and the polymer. As a result, when using the poly-surface agent for oil driving, a smaller concentration adjustment can effectively modify the viscosity, resulting in a significant reduction in the amount of polymer required.

The viscosity tests were carried out by subjecting the polymers to shearing for 20 s using a stirring device. The viscosity measurements were taken before and after shearing for three separate polymerizations. The findings presented in Figure 4b demonstrate that the polyepitaxial agent exhibits a notable retention of viscosity, consistently surpassing 70% and even exceeding 90% at higher concentrations. In contrast, the salt-resistant polymer and medium-scored polymer exhibit viscosity retention ranging from 60% to 80%. These results highlight the capacity of the polyepitaxials’ spatial network structure to recover autonomously after experiencing shear damage. The intermediate polymer demonstrated a marginally higher ability to maintain viscosity at low concentrations, followed by the polymeric surfactant and, ultimately, the salt-tolerant polymer. The viscosity retention rate of poly-epimers at low concentrations is similar to that of polymers, primarily attributed to the decreased viscosity of poly-epimer solutions in low-concentration systems, the dispersed molecular distribution, and the limited structural degradation caused by shearing effects.

However, the bonding of the polysurfactant exhibits a restricted effect, whereas the pivotal association (at a concentration of approximately 800 mg/L) of the polysurfactant also manifests a negligible influence. This association serves to illustrate the inherent ability of the intrinsic viscosity to endure shear forces. With an escalating concentration, the rate of viscosity preservation gradually escalates, implying that once the critical viscosity is achieved, the structural viscosity arising from bonding substantially surpasses the decline in intrinsic viscosity induced by shear impairment. The relationship between viscosity retention rate and viscosity in mid-part polymers is inversely proportional, with lower viscosity levels corresponding to higher retention rates. Moreover, the retention rate remains relatively stable as polymer concentration increases, attributed to the dispersed distribution of molecules at lower concentrations and the limited occurrence of shear damage. However, as the concentration further increases, the probability of shearing rises, resulting in a decline in the viscosity retention rate. In cases of elevated concentration, the shearing effect predominantly affects structural impairment, while the rate of viscosity retention demonstrates a tendency toward stability. Despite their strong viscosity-enhancing properties, salt-tolerant polymers exhibit shear resistance similar to polymers with moderate ratings.

**Figure 5:**
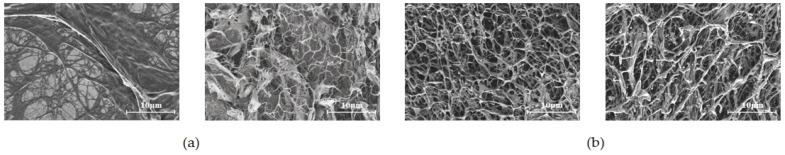
Comparison results of morphology before and after shearing: (**a**) Left before polyepoxide shear, right after polyepoxide shear; (**b**) The left side is before shearing, and the right side is after shearing of the centered polymer.

The comparison of molecular microstructure before and after shearing is depicted in Figure 5. The spatial network structure of the multi-epitaxial agents undergoes a minor deterioration but still maintains distinguishable spatial patterns, lamellar layer loads, and highly refined filament structures. Importantly, a network structure emerges following shearing, as illustrated in Figure 5a, indicating the existence of a noticeable gradient of damage within the spatial network structure of the two states. The formation of the network structure relies on the existence of a low shearing force and a favorable network structure. The susceptibility of this structure to temporary damage is counterbalanced by its intermolecular association structure’s capacity for self-restoration over time. In contrast, the medium-scoring polymer displays multiple sheared chain ports, as illustrated in Figure 5b, indicating a more pronounced linear structure.

The investigation focused on the hydrodynamic properties of oil-repellent systems incorporating poly-surfactants. The hydrodynamic size of the polysurfactant was determined through a microporous membrane detection method with varying pore membrane radii [33,34]. The hydrodynamic sizes of various systems at varying concentrations are displayed in Table 2. It was observed that, within polymer solutions of equivalent concentrations, the hydrodynamic size of the polysurfactant was significantly greater than that of the salt-resistant polymer and notably larger than that of the middle-partitioned polymer. Furthermore, the hydrodynamic characteristic size of the surface-polymerizing agent demonstrates a discernible inflection point as the concentration rises, suggesting a correlation with the critical association concentration of the surface-polymerizing agent molecules. Based on the analysis of viscosity increase, the concentration can be inferred to be approximately 800 mg/L. The hydrodynamic dimensions of the middle-fractionated polymers consistently measure below 0.75 μm. Furthermore, the growth remains relatively stable and gradually decelerates with varying concentrations. This observation implies that the long-chain intertwined coil-like structure demonstrates stable flow properties.

To achieve specific viscosity values of 30 mPa·s, 60 mPa·s, and 120 mPa·s, the viscosity of the polymer solution was manipulated accordingly. The hydrodynamic characteristic sizes of three different polymers were determined at varying temperatures, taking into account the impact of viscosity. When comparing the hydrodynamic characteristic sizes under the same viscosity conditions, it was observed that at a low viscosity of 30 mPa·s, the polysurfactant and intermediate polymer exhibited similar sizes, which were larger than that of the salt-tolerant polymer. At a viscosity of 60 mPa·s, the hydrodynamic characteristic size of the polysurfactant exceeded that of the intermediate polymer. Following this, when the viscosity reaches 120 mPa·s, the hydrodynamic characteristic size of the polysurfactant surpasses that of both the intermediate polymer and the salt-tolerant polymer. These results are displayed in Table 2.

The hydrodynamic characteristic size of polymers is predominantly affected by the viscosity and concentration of the solution. The results presented in Table 2 suggest that the molecular structure of the polysurfactant readily forms and establishes a continuous spatial network structure, even before reaching the critical association concentration. This phenomenon is concomitant with an elevation in viscosity, hydrodynamic attributes, and overall dimensions. These findings provide evidence that the hydrodynamic characteristic scale of polymers is primarily governed by the spatial configuration established by the molecules within the solution. The size of the molecular network structure predominantly impacts its magnitude, whereas the viscosity of the solution does not exhibit a direct correlation. The viscosity of the solution is determined by the size of fluid dynamics and is a result of the hydration and interconnection of polymer molecules, leading to the creation of a spatial structure. The alignment between hydrodynamic size and concentration allows for the modulation of viscosity by adjusting the concentration, thereby controlling fluidity in the application of surface polymerizers. Concurrently, the utilization of concentration can be employed to efficiently control a wider hydrodynamic radius, thereby facilitating the systematic extraction of oil reservoirs.The capacity to regulate plugging and oil displacement is gradually achieved by gradually decreasing the concentration. It is precisely due to this mechanism that surface polymerization agents demonstrate adaptive characteristics for regulation and flooding.

### 3.3. Polysurfactant Oil Repellent Characteristics

The aim of this research was to examine the microscopic oil-repellent characteristics of polysurfactants. To accomplish this, a microfluidic chip (Daqing Oilfield, Daqing, China) was constructed utilizing a naturally occurring core cast thin section from the mesoporous-medium-permeable reservoir in the Daqing oilfield, which displayed desirable physical attributes, including a uniform distribution of pore throats and strong connectivity. The experimental oil employed in this investigation was a simulated crude oil with a viscosity of 9.8 mPa·s at a temperature of 45 °C.

This study employs the utilization of a constant pressure drive with varying injection pressures under isoviscous conditions. The injection pressures were determined based on the fact that the maximum injection pressure during the field test will not exceed the rupture pressure, and the blank water drive phase is typically depressurized to a range of 1/3–1/2 of the rupture pressure. The purpose of establishing these injection pressures, as indicated in Table 3, was to enable a comparative analysis of the viscoelastic oil driving or washing effects demonstrated by the three polymer systems.

**Figure 6:**
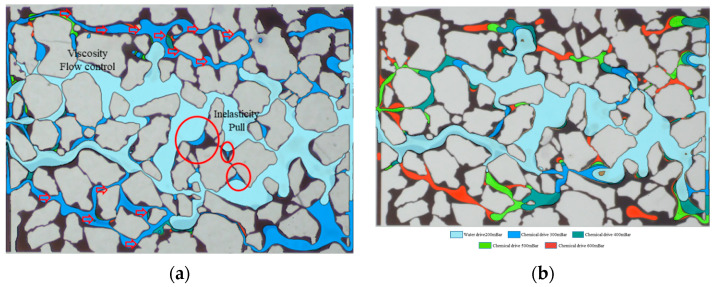
Identification diagram of different stages of microscopic pore turnover players: (**a**) Polysurfactant; (**b**) Polymer with a neutral parting.

The analysis of wave area modification before and after the oil drive on the two-dimensional model allows for the assessment of enhanced recovery levels at different pressure levels. The findings are displayed in Table 3. It is noteworthy that all three polymers contribute to an increase in ripple volume beyond what is achieved solely through water drive. Specifically, the poly-surfactant exhibits a 35.52% improvement in recovery compared to water drive at 600 mBar, surpassing the salt-resistant polymer (30.85%) and the medium-split polymer (25.09%). The current investigation utilized image characterization methodologies to distinguish different phases of crude oil movement within the model, employing distinct color coding. The findings of this analysis are visually depicted in Figure 6. It is worth noting that the poly-surface agent exhibited a significant enhancement effect on recovery within the pressure range of 300–500 mBar. Nevertheless, this enhancement effect becomes insignificant once the pressure surpasses the threshold of 500 mBar, indicating that the agent’s capacity to expand the wave volume has reached its maximum limit.

The poly-surfactant demonstrated a notable flow control effect during the microscopic oil driving process, leading to a 29.77% increase in recovery enhancement during the chemical driving stage when the replacement pressure was raised by 1.5 times (300 mBar). Moreover, the poly-surfactant exhibited a distinct elastic pulling effect on blind end-like residual oil, indicating its superior viscoelastic oil-repellent properties. The polymer displayed a prolonged duration of action and consistently enhanced recovery across all pressure stages. However, the overall level of enhancement is relatively minimal, indicating the need for further optimization of the viscoelastic oil-repellent effect of the polymer under increased differential pressure during replacement.

Furthermore, the field test, constrained by limitations imposed by the oil formation, demonstrates that a lower injection pressure promotes the superior expansion of the wave and volume effect, as well as the elasticity of the oil-repellent effect, thereby offering greater advantages. A reduced injection pressure proves to be more advantageous for field testing as a consequence of the constraints imposed by the fracture pressure of the reservoir. In comparing salt-resistant polymers, their linear molecular structure confers an advantage in accommodating larger pore throats, thereby enhancing their ability to expand waves and volumes in comparison to other polymers. Nevertheless, these polymers exhibit inferior elasticity and interfacial activity when compared to poly-surfactants, resulting in a diminished capacity to repel oil at lower pressures.

The emulsification behavior of polysurfactants is impacted by the existence of surface-active functional groups in the surface-polymerizing agent, leading to the formation of a crude oil emulsion. The size of the emulsion particles, their rheological properties, and the patterns of migration provide insights into the emulsification behavior demonstrated by the surface-polymerizing agent. The utilization of emulsification carrying and emulsification plugging effects during the emulsification process can significantly improve the outcome of oil recovery.

**Figure 7:**
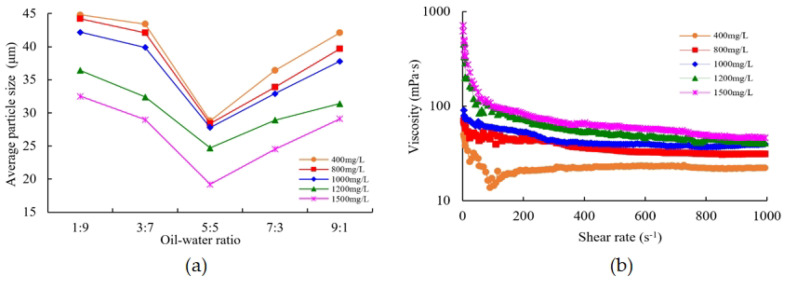
(**a**) The average particle size curve of emulsion with different concentrations of polymer surfactant under different oil-water ratio conditions. (**b**) Emulsion rheological curve (oil-water ratio 5:5).

The emulsion’s particle size plays a significant role in determining its stability and fluidity. As illustrated in Figure 7a, the average particle size of the polysurfactant emulsion varies depending on the oil-to-water ratio. The results suggest that the polysurfactant emulsion typically has a larger average particle size, with a particle size distribution ranging from 10 μm to 50 μm. When the oil-water ratio is held constant, an elevation in the concentration of the surface-polymerizing agent results in a reduction in the mean particle size of the emulsion. This finding implies that increasing the concentration of the surface-polymerizing agent has the potential to enhance the stability of the emulsion. As an emulsion is a system characterized by a substantial phase interface, it inherently tends to undergo spontaneous coalescence of its emulsion beads in an effort to minimize the energy associated with the phase interface.

The present study demonstrates that the increased viscosity of the external phase has the capacity to reduce the occurrence of collisions and the speed of merging between small droplets, thereby facilitating the stability of the emulsion. Additionally, the polysurfactant plays a dual role: firstly, it lowers the interfacial tension of the oil-water system by introducing active functional groups; secondly, it enhances the system’s viscosity through the steric hindrance effect. When the concentration of the critical association exceeds 800 mg/L, the emulsion displays a decrease in water separation and demonstrates a relatively stable behavior. Under the same concentration of polysurfactant, the average particle size of the emulsion initially decreases and then increases as the oil-water ratio increases. It is worth noting that the emulsion achieves its smallest average particle size when the oil-water ratio is 5:5.

In conclusion, the improved stability of the emulsion is associated with a reduction in the size of the emulsified particles. The results obtained from constant-rate mercury injection analysis of well cores in the Sazhong Development Zone suggest that cores with an absolute permeability within the range of 500–2000 × 10^−3^ μm^2^ demonstrate an average throat diameter ranging from 7.60 μm to 19.32 μm. As a result, the formation of a stable emulsion within the oil layer can effectively control its fluidity and, by means of the Jiamin effect, promote an “elastic” displacement of emulsified oil droplets during flow [35].

The behavior law of the polysurfactant emulsion during flow is characterized by the change rule of its rheological properties. Figure 7b illustrates the rheological property change curve of the emulsion at an oil-water ratio of 5:5. The findings indicate that the emulsion exhibits noticeable shear-thinning properties and displays typical pseudoplastic fluid behavior. The viscosity of the emulsion is influenced by the combined effect of the polysurfactant’s viscosity and the emulsion’s viscosity, as demonstrated by a double superposition effect [36].

At low shear rates, the higher the concentration of the polysurfactant system, the greater the contribution of the viscosity of the dispersion medium in the emulsion system, the higher the viscosity of the emulsion system, and the higher the viscosity of the system above the critical association concentration of the polysurfactant. At low shear rates, an increase in the concentration of the polysurfactant system results in a greater contribution of the dispersion medium’s viscosity in the emulsion system.

As a result, the viscosity of the emulsion system is observed to rise, particularly when the concentration of the polysurfactant surpasses the critical association concentration. From the aforementioned examination of emulsified particle size and rheological properties, it can be deduced that the emulsion system showcases viscosity and displays viscoelastic characteristics while undergoing oil displacement, which is marked by elastic deformation and subsequent recovery as it traverses the pore throat.

**Figure 8:**
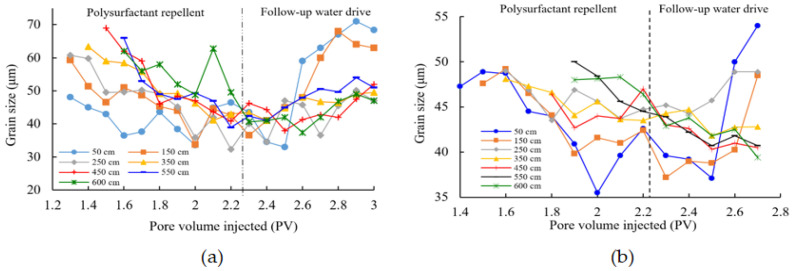
Curves of mean emulsion particle size variation at different sampling locations in hypertonic and hypotonic cores: (**a**) Results of emulsified particle size of poly-surfactant in high permeability cores; (**b**) Low-permeability core poly-surface agent emulsion particle size results.

A 600 cm parallel sand-packing pipe model was utilized to conduct oil displacement experiments. Samples were collected from multiple sampling points to analyze the spatial distribution and migration patterns of the resulting emulsion. Figure 8 depicts the average variation curves of emulsion particle size at different positions within high-permeability cores and low-permeability cores.

The average particle size at the hypertonic end demonstrates an initial decrease followed by an increase, while the average particle size of the emulsion exhibits notable fluctuations. In contrast, the average particle size at the hypertonic end exhibits a relatively stable pattern, and the distribution of particle sizes remains uniform throughout the entirety of the process. It is worth noting that the particle size undergoes more significant alterations at the high seepage end, specifically at 450 cm, upon the introduction of the pore volume of the poly-surfactant. This observation implies a heightened tendency for the formation of an emulsion system at this specific location. Based on the results of the field test, it can be inferred that a particle size below 45 μm corresponds to a medium emulsification strength, whereas a particle size exceeding 45 μm indicates a weak emulsification strength. Additionally, as the sampling point moves away from the inlet end, the duration of medium emulsification decreases.

The data presented in Figure 8a suggest that the stability of the poly-surfactant emulsion is influenced by external factors such as the oil-water ratio and the concentration of the poly-surfactant. A higher concentration of the poly-surfactant and a more balanced oil-water ratio offer benefits in attaining an emulsion characterized by smaller and more stable particle sizes. Nevertheless, it is important to acknowledge that the variability in the average particle size of the resulting poly-surfactant emulsion is influenced by fluctuations in the oil-water ratio and the concentration of the poly-surfactant at the sampling points.

During the polysurfactant substitution process, there is an increase in the size of emulsion particles, resulting in the observation of light emulsification and medium emulsion oil-in-water types. The medium emulsification of each sampling point consistently experiences a delay in time, while the extent of emulsification beyond the medium level demonstrates a decreasing trend. The duration of emulsification in the high permeability layer spans from the introduction of polysurfactant to 0.2 PV to 0.8 PV. The duration of medium emulsification varies from the introduction of polysurfactant to 0.5 PV, with a maximum duration of 1.1 PV. The commencement of the low permeability layer aligns with the introduction of polysurfactant and extends to 0.5 PV. Subsequently, the duration is reduced from 0.5 PV to 0.8 PV at the initiation of polyepoxide injection and further decreased from a maximum of 0.8 PV to 0.4 PV during mid-term emulsification after polyepoxide injection. The statistical findings are presented in Table 4.

The emulsification of the surface-polymerizing agent takes place after 0.2 PV in the long-fill sand pipe model. Due to the potential for improved oil recovery through emulsification, the microfluidic experimental chip’s small size and limited presence of disturbance units pose challenges in achieving direct emulsification within it. Consequently, the displacement of production fluid within the sand-packed pipe model is utilized to replicate the polymer emulsion flooding process. During the course of the experiment, it was observed that the oil generated within the sand-packing pipe model displaces the residual oil within the microfluidic model. As a result, the investigation of emulsification juxtaposes the results obtained after water flooding with those obtained after water flooding to determine the influence of the emulsion on improving recovery.

**Figure 9:**
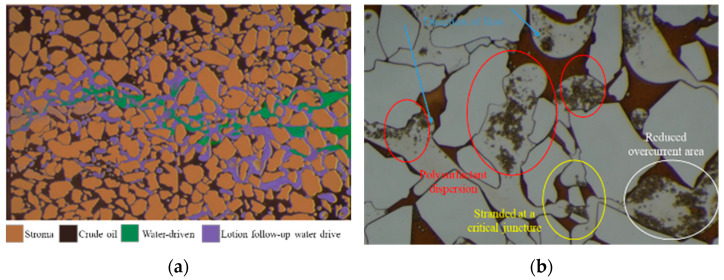
At different stages of the process, the effects of polymer surfactant on enhanced oil recovery and emulsion carrying and plugging in microfluidic chips were compared: (**a**) Comparison results of enhanced oil recovery at different stages; (**b**) Emulsion carrying and emulsion blockage.

The effects of enhanced oil recovery at different stages are illustrated in Figure 9a. The results suggest that the polysurfactant emulsion demonstrates a strong capability for enhanced oil recovery. Under an injection pressure of 300 mBar, the polysurfactant and emulsion work together synergistically to enhance oil recovery, with the adsorbed and retained polysurfactant playing a contributing role. Subsequent water flooding further increases the ultimate enhanced oil recovery to 34.8%, surpassing the 29.77% achieved by the pure polysurfactant by 5.03%.

During the emulsion flooding process, the expansion of the swept volume can be observed through the mechanism illustrated in Figure 9b. The emulsion demonstrates a significant effect in carrying out emulsification. Visual observations indicate that the emulsion predominantly adopts an oil-in-water (O/W) emulsification type. This is attributed to the O/W emulsion having a lower viscosity compared to the oil phase, yet higher than that of the water phase, resulting in improved fluidity. Consequently, when the emulsion aggregates, it is compelled to enter the remaining oil present in the continuous sheet. Additionally, the phenomenon of emulsification and plugging effects is demonstrated [37]. Following the formation of the emulsion, adsorption occurs on the surface of the rock, particularly in areas with lower flow rates, resulting in the adsorption of the polysurfactant. Consequently, this phenomenon leads to a reduction in the flow area, ultimately achieving the desired effect.

Furthermore, it is observed that the emulsion droplet exhibits a Hernes step phenomenon when encountering smaller pore throats. If the displacement pressure difference does not exceed the necessary pressure difference for emulsion passage, the emulsion droplet will exhibit noticeable bridging phenomena, hindering its movement through the pore throat. This obstruction prevents the subsequently injected fluid from flowing, causing it to change direction and ultimately resulting in an expansion of the swept volume. This phenomenon is likely to occur during the surface polymer flooding process, especially when the central region of the oil layer nears the production end. As the displacement pressure differential decreases and the seepage velocity declines, a significant amount of oil-in-water (O/W) emulsion will accumulate within the pore throats, leading to the creation of an emulsified oil barrier. The results of the oil displacement test conducted on the 600 cm parallel sand-packing pipe model indicate that accumulation is likely to occur at the 450 cm position, which corresponds to approximately three-quarters of the distance between the injection and production wells.

**Figure 10:**
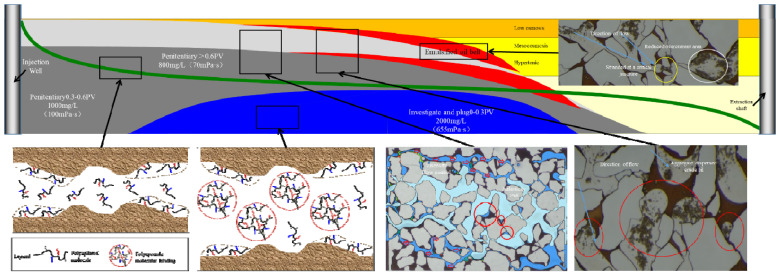
Poly-surface agent “adjusting, driving and blocking” comprehensive digging potential mode.

The comprehensive potential tapping mode of “regulating, flooding, and plugging” is accomplished by employing a surface-polymerizing agent. This agent exhibits diverse functionalities, including association viscosity, high adsorption retention, and robust fluidity control. As a result, it is capable of achieving the multiple effects of “adjustment, flooding, and plugging” using a solitary agent. Figure 10 depicts the schematic diagram of this comprehensive potential tapping mode during the entirety of the surface agent polymerization process. During the initial phase of chemical flooding, a polysurfactant with a high concentration is utilized. This polysurfactant exhibits the ability to be adsorbed onto the negatively charged sandstone surface that is devoid of oil, owing to its association viscosity. Furthermore, the modification of the interface leads to a reduction in the pore throat radius of the high-permeability layer. This modification effectively controls the occurrence of plugging within the high-permeability layer, as only a minimal amount of association molecules with a large diameter are able to enter the low-permeability layer.

Concurrently, the high-permeability layer effectively manages fluidity by employing high viscosity within its structure. As a result, the injection pressure gradually increases until it reaches the initiation pressure of the thin, less permeable layer. Once the plugging is suitably controlled, a lower concentration of surface-polymerizing agent can be utilized for the flooding process. At this point, the initiation pressure of the less permeable layer has been achieved. Upon the introduction of the surface-polymerizing agent, both the low-permeability layer and the high-permeability layer undergo simultaneous flushing, while the viscoelastic oil displacement induced by the surface-polymerizing agent also aids in the flooding process. As the oil saturation in the central region of the oil layer rises, the emulsification carrying effect is further enhanced, leading to the dispersion and transportation of crude oil through an oil-in-water emulsion.

After the generation of the emulsion, a significant accumulation of the emulsion will occur at the pore throat in the area with a decreased flow rate, leading to the development of an emulsified oil wall and subsequent occurrence of an emulsified plugging effect. As a result, the emulsified oil wall assumes an additional role in regulating fluidity, thereby facilitating a flow-around effect within the displacement system. This process will be repeated through the continuous introduction of the polysurfactant.

The emulsified oil wall will gradually form within the reservoir, undergoing a transition from regions of high permeability to regions of low permeability due to the displacement of oil by the polysurfactant. As the resistance of the high permeability layer increases, the liquid flow will redirect towards another low permeability layer, leading to the formation of a high resistance emulsified oil zone at the interface between oil and water. Consequently, multiple zones of emulsified oil enrichment will ultimately be established. The development of these emulsified oil zones shares similarities with the repeated implementation of emulsion profile control techniques in the oil reservoir [38,39]. As a result, the injection capacity will demonstrate minimal fluctuations within a designated time period, while the production capacity will undergo a substantial decrease. Simultaneously, the water cut will decrease, and the pressure within the middle section of the oil layer will rapidly increase, necessitating the prompt initiation of fracturing induction [40].

The application of modulation, flooding, and plugging techniques, specifically with the assistance of polymer surface agents, demonstrates significant potential for augmenting oil recovery after polymer flooding. This indicates the progression towards the quaternary phase of oil recovery. Given the emergence of dominant channel post-polymer flooding, the implementation of a high-viscosity displacement system becomes crucial for effectively controlling plugging throughout the entirety of the oil recovery enhancement procedure. Concurrently, in order to sustain injection capacity throughout the procedure, it is imperative to employ a concentration-reducing and speed-enhancing injection technique to amplify the swept volume while guaranteeing the injection rate.

Consequently, due to prolonged and insufficient water circulation, the subsequent production phase after polymer flooding faces difficulties arising from the prevalence of dominant channels, rendering it more arduous to influence production solely through modifications at the injection phase. A consensus has been reached in the field of ASP flooding, recognizing the improvement in emulsification and recovery rates [41,42]. Therefore, incorporating a rational emulsification plugging approach in the design process of polymer flooding can effectively divert liquid flow and facilitate emulsification solubilization. These suggestions for post-polymer flooding adjustments have successfully embraced the concept of advancing post-polymer flooding through the use of polymer surface agents.

### 3.4. Field Test Effect of Polymer Surface Agent “Blocking, Regulation and Flooding” after Polymer Flooding

The research encompassed the development of injection techniques and the assessment of dynamic variations in the “blocking, regulating, and flooding” injection of a polymer surface agent subsequent to polymer flooding. The initial field experiment of polymer surface agent flooding was executed in the Duandong area of the North 1 District in the Daqing Oilfield. The experiment comprised 18 injection wells, 28 production wells, a five-point well pattern, and injection-production well spacing of 106 m and 150 m. During the field experiment, the polymer surface agent was introduced into the system with a high-viscosity, low-speed injection method at the initial stage of betting (0–0.25 PV) while ensuring careful regulation of the pressure increase. After the completion of the plugging adjustment, it is advisable to decrease viscosity and increase injection speed during the subsequent flooding stage (0.25 PV-end) to ensure a consistent injection pressure.

**Figure 11:**
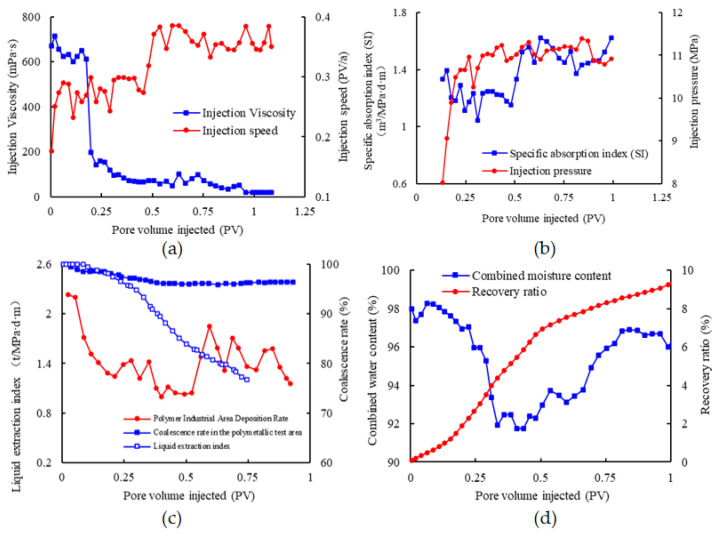
Site design and recovery results of post-polydrive poly-surface-agent “plug-and-regulate” drives: (**a**) Variation curves of injection viscosity and injection rate in the test area; (**b**) Variation curves of specific water absorption index and injection pressure in the test area; (**c**) Results of liquid extraction index and stocking rate in the test area; (**d**) Comprehensive water content and recovery rate results of the test area.

The maintenance of a stable injection pressure level in the injection well is achieved through the implementation of distinct injection techniques. Through the utilization of the associative viscosity properties of the polysurfactant, a methodical strategy has been developed to progressively diminish viscosity and augment velocity throughout the entirety of the procedure. In the preliminary test area, the concentration of injection was established at 2000 mg/L, accompanied by an initial injection viscosity of 655 mPa·s at the wellhead. Subsequently, the injection viscosity experienced a gradual reduction over the duration of the process. After a swift decline in water cut, the injection concentration was adjusted to 1016 mg/L at 0.25 pore volume (PV). The viscosity of the wellhead injection was quantified at 84.5 mPa·s, and the concentration was subsequently modified to maintain an appropriate injection rate in accordance with the injection pressure. The changes in injection viscosity and injection rate over the entire process are illustrated in Figure 11a. Additionally, Figure 11b displays the results of the specific water absorption index and injection pressure for the injection well based on the injection pore volume.

The injection pressure displayed a gradual increase in the initial stage and subsequently maintained stability in the later stage, indicating the influence of profile adjustment. The specific water absorption index, initially recorded as 1.41 m^3^/MPa·d·m for the polymer injection surface agent, rose to 1.74 m^3^/MPa·d·m after implementing adjustments to decrease concentration and expedite the process. This increase signifies a strong injection capability throughout the entirety of the process. Simultaneously, the production ratio of thin and low-quality oil layers underwent a significant increase from 57.8% to 77.2%.

The study investigated the evolving changes and effects of polymer surface agent production subsequent to polymer flooding. The liquid production index at the production site displayed a gradual decline, as illustrated in Figure 11c. More specifically, the liquid extraction index in the experimental area decreased from an initial value of 2.2 t/d·MPa·m to a minimum of 1.0 t/d·MPa·m, indicating a reduction of 54%. Moreover, this decline exhibited secondary decline characteristics. Following the introduction of the low-viscosity surface-polymerizing agent through injection, a consistent decrease in the liquid production index was observed, indicating that the reduction in liquid production capacity cannot be solely attributed to the viscosity of the surface-polymerizing agent. While no significant change in injection capacity was noted at the injection point, a significant decline in liquid production indicated the occurrence of a noticeable phenomenon of emulsification plugging at the production point.

Concurrently, the comparative analysis of the fluid accumulation ratio suggests that the test area designated for polymer surface agents demonstrates a higher propensity for chemical agent adsorption in contrast to the industrial area for polymers. This observation highlights the strong adsorption and retention capabilities of the polymer surface agent, which aligns with the results obtained from laboratory experiments. A comprehensive investigation of both the injection and production aspects reveals that the rapid accumulation rate exhibits characteristics of interfacial modification and adsorption. The sustained injection pressure serves as an indicator of the polymer surface agent’s strong flow capacity, whereas the decline in the fluid production index validates the occurrence of emulsification and plugging. Subsequently, a moderate level of fracturing effect is employed, leading to the retrieval of fluid volume and a rise in the proportion of emulsified oil generated.

The Duandong polysurfactant test area in the North 1st District successfully implemented the polymer surface agent “regulation, flooding, and plugging” supporting adjustment technology for a period of 5 years following polymer flooding. During the chemical flooding phase, the injection rate was 0.2 PV/a, resulting in an annual oil recovery rate of 1.88%. The maximum reduction in water cut reached 6.09%, and the cumulative oil production reached 119,700 tons. The stage recovery rate was 11.02%, and the overall recovery rate reached 64.22%. The current level of enhanced oil recovery is 9.4%, as indicated through a thorough analysis of the water cut, which stands at 97% (as illustrated in Figure 11d). It is projected that the final enhanced oil recovery will reach 10.85%. To assess the economic feasibility, the cash flow method is utilized, which considers drilling depreciation and fracturing measures in accordance with the current oil price. The input–output ratio is determined to be 1:3.86, suggesting favorable economic advantages.

## 4. Conclusions

(1) The polysurfactant is subjected to block copolymerization with AMPS and functional monomers, leading to the integration of diverse functional groups and a subsequent augmentation of interfacial activity. The resultant molecular chains manifest a dense, interconnected configuration, thereby establishing an extensive network conformation. The inclusion of multi-branched structures notably amplifies the association effect. The critical concentration for the association is estimated to be around 800 mg/L, at which juncture the polysurfactant exhibits a viscosity retention rate surpassing 90% subsequent to shearing. The ability to manipulate changes in concentration facilitates control over the characteristic size of hydrodynamics. Additionally, the gradual reduction in concentration enables the achievement of step-by-step adjustments in plugging and flooding effects, thereby showcasing the self-adaptive qualities of single-agent adjustment, flooding, and plugging.
(2) The polysurfactant demonstrates the capacity to emulsify crude oil, leading to the formation of emulsified oil particles ranging in size from 10 μm to 50 μm. The resulting emulsion exhibits characteristic pseudoplastic fluid behavior and effectively regulates fluidity through the Jamin effect. Emulsification is more prone to transpire in areas with elevated oil saturation, predominantly resulting in an oil-in-water (O/W) emulsion. At a microscopic scale, emulsification facilitates improved oil recovery through emulsion transport and emulsion plugging mechanisms.
(3) The polyepitaxial agent exhibits a range of functionalities such as bonding viscosity, high adsorption and retention, robust fluidity control, emulsification carrying, and blocking. These attributes are in line with the concept of “adjusting, driving, and blocking” to enhance post-polymer drive recovery in the Daqing Oilfield. This study effectively showcases the feasibility of the utilizing poly-surface agent in “adjusting, driving, and plugging” recovery enhancement technology through the implementation of an initial field test in the North Area of the Daqing Oilfield. The findings suggest that this approach has the potential to yield a substantial increase of 10.85% in the recovery rate, accompanied by a favorable input–output ratio of 1:3.86, thereby generating significant economic advantages.

## Figures and Tables

**Table 1:** Comparison of spatial structures of three polymers.

Sample	Polysurfactants	Medium-Scoring Polymers	Salt-Resistant Polymers
Chain type	Short-Branch	Long-Roll	Long-straight
Network structure	Inter/intramolecular bonding, cross-linking	Long chains entangled with each other	Long chains entangled with each other
Network branching	Side chain cross-linking	Electrostatic repulsive stretching of molecular chains	Electrostatic repulsive stretching of molecular chains
Distribution of molecular chains	Coarse, Uneven	Fine, Uniform	Coarse, Uniform

**Table 2:** Hydrodynamic characterization and static adsorption results for three polymers.

Test Items	Concentration (mg/L)		Polymer Type		
			Polymeric Agent	Anti-Salt Polymers	Polymer with a Neutral Parting
Measurement results of hydrodynamic characteristic size of oil displacement system (μm)	500		0.65	0.45	0.3
	700		0.83	0.5	0.45
	1000		1.2	0.65	0.60
	1500		3	0.75	0.68
	2000		4.5	1.2	0.75
Comparison of hydrodynamic characteristic dimensions of three polymers under is viscous conditions (μm)	Viscosity (mPa·s)	Polymer type			
	30	Concentration (mg/L)	500	500	1500
		Hydrodynamic feature size (μm)	0.65	0.45	0.6
	60	Concentration (mg/L)	700	700	2000
		Hydrodynamic feature size (μm)	0.93	0.5	0.75
	120	Concentration (mg/L)	800	1000	3000
		Hydrodynamic feature size (μm)	1.2	0.65	0.9

**Table 3:** Enhanced oil recovery at different stages of three polymers.

Replacement Type	Injection Pressure (mBar)	Recovery Rate at Different Pressures (%)		
		Polysurfactants	Salt-Resistant Polymers	Medium Fraction Polymers
Water drive	200	41.46	40.54	38.15
Chemical drive	300	71.23	68.61	48.67
	400	73.50	70.26	55.39
	500	76.98	71.27	61.15
	600	76.99	71.39	63.24
Ultimate recovery enhancement (%)		35.52	30.85	25.09

**Table 4:** The timing and duration of medium emulsification at different positions of the core.

Pressure Point (cm)	Conversion to Injection Well Distance Ratio (%)	High Permeability		Low Permeability	
		Timing of Mid-Emulsion Generation (PV)	Duration of Mid-Emulsification (PV)	Timing of Mid-Emulsification (PV)	Duration of Mid-Emulsification (PV)
50	8	0.2	1.1	0.5	0.8
150	25	0.7	0.7	0.6	0.7
250	42	0.8	0.5	0.6	0.7
350	58	0.9	0.5	0.7	0.6
450	75	0.9	0.5	0.7	0.6
550	92	1	0.4	1	0.5
600	100	1.1	0.4	1.1	0.4

## Data Availability

Data are contained within the article.
